# Childhood Household Dysfunction, Social Inequality and Alcohol Related Illness in Young Adulthood. A Swedish National Cohort Study

**DOI:** 10.1371/journal.pone.0151755

**Published:** 2016-03-18

**Authors:** Karl Gauffin, Anders Hjern, Bo Vinnerljung, Emma Björkenstam

**Affiliations:** 1 Centre for Health Equity Studies, Stockholm University/Karolinska Institutet, Stockholm, Sweden; 2 Clinical Epidemiology/Department of Medicine, Karolinska Institutet, Stockholm, Sweden; 3 Department of Social Work, Stockholm University, Stockholm, Sweden; 4 Department of Public Health Sciences, Karolinska Institutet, Stockholm, Sweden; 5 Department of Community Health Sciences, Fielding School of Public Health and California Center for Population Research, University of California Los Angeles, Los Angeles, California, United States of America; California Northstate University College of Medicine, UNITED STATES

## Abstract

The aim of this paper is to estimate the cumulative effect of childhood household dysfunction (CHD) on alcohol related illness and death later in life and to test the interaction between CHD and socioeconomic background. The study utilised Swedish national registers including data of a Swedish national cohort born 1973–82 (n = 872 912), which was followed from age 18 to 29–40 years. Cox regression analyses were used to calculate hazard ratios (HR) for alcohol related illness or death in young adulthood. The CHD measure consisted of seven indicators: parental alcohol/drug misuse, mental health problems, criminality, death, divorce, social assistance, and child welfare interventions. Childhood socioeconomic position (SEP) was indicated by parental occupational status. Outcomes were alcohol related inpatient hospital care, specialised outpatient care or deaths. Using the highest socioeconomic group without CHD experience as a reference, those in the same socioeconomic group with one indicator of CHD had HRs of 2.1 [95% CI: 1.7–2.5], two CHD indicators 5.6 [4.4–7.1], three or more indicators 9.4 [7.1–12.4] for retrieving inpatient care. Socioeconomic disadvantage further increased the risks–those with low socioeconomic background and three CHD indicators or more had a HR of 12.5 [10.9–14.3]. Testing for interaction suggests that the combined HRs deviates from additivity [Synergy index: 1.6, 95% CI: 1.4–1.9]. The results for outpatient care were similar, but not as pronounced. In conclusion, this Swedish national cohort study shows that childhood household dysfunction is strongly and cumulatively associated to alcohol related illness later in life and that it interacts with socioeconomic disadvantage.

## Introduction

Intergenerational social mobility, or the potential of children to reach a higher socioeconomic position (SEP) than their parents, is relatively high in Sweden. Compared to a child born in the US, it is far more likely that a Swedish child will successfully pursue the ‘American dream’–starting with nothing and ending up with plenty [1]. However, also in Sweden, family background is a major predictor of SEP in adulthood [2]. In a previous study, it was shown that alcohol related illness is more common in groups with a low socioeconomic background [3]. This result is contrasted by a systematic review on the topic and the Swedish national public health survey, as they show no association between SEP in childhood or adulthood and alcohol consumption or alcohol misuse [4, 5]. This leads us to argue that the overrepresentation of alcohol related illness in groups with low socioeconomic background is not related to higher alcohol consumption (exposure), but rather to increased vulnerability to the adverse effects of alcohol [6]. Over the life course of a person growing up in a low SEP household, a number of factors adding to this vulnerability will be likely to accumulate, including lack of material resources, low school performance, low educational level, low-paid stressful jobs, occupational hazards and poor general health [7, 8]. In contrast, groups growing up under privileged socioeconomic circumstances will be likely to experience a number of advantageous factors adding up to a buffer that may compensate for high alcohol consumption later in life. High income and education of the parents will promote success in school, leading to higher education and well-paid jobs with good benefits. The risk for poor general health and well-being will be lower in these groups, as will the risk for adverse health effects of alcohol [9].

Other studies investigating early life risk factors for alcohol related illness later in life focus on the effects of adverse childhood experiences (ACE) [10–13]. A team of American researchers led by Felitti and Anda [14, 15] have defined ACE using three sub-measures: abuse, neglect and childhood household dysfunction (CHD). Researchers involved in the American ACE studies have used retrospective survey data for over 17,000 individuals to produce a large number of articles on the relationship between ACE and adult health outcomes such as depression, obesity, suicide, smoking and alcoholism [16–20]. The studies have found that not only are ACE very common (only 1/3 of the study participants had *no* ACE), but also that their connection to health problems later in life is strong and cumulative [15]. Studies using other data and focusing on specific ACE sub-measures have been able to confirm these results and discuss a range of potential mechanisms explaining the relationship between ACE and adult health. Whereas some studies emphasise the biological and psychological effects of stress and trauma, others focus on more sociological approaches taking the interaction between ACE and socioeconomic circumstances into consideration [21–23].

The aim of this paper is to estimate the effect of household dysfunction in childhood on alcohol related illness and death later in life. In line with previous research, we hypothesise that experiencing CHD will have a cumulative effect on the risk for developing alcohol related illness. Additionally, we want to estimate the interaction between socioeconomic background and CHD.

## Methods

Ethical approval was granted by The Regional Ethical Review Board (Regionala etikprövningsnämnden) in Stockholm, Sweden, with approval number dnr 2009/2027-31/5; (including complimentary ethic approvals with dnr 2012/657-32 and 3013/1058-32). Swedish population registers include a large number of social, educational and health related indicators and provide researchers in social epidemiology with excellent data for longitudinal population based studies. Every Swedish resident is given a unique personal identity number (PIN) that stays with the individual from time of birth or immigration to death. The PINs enable record linking, which makes it possible to follow a person over time in multiple population registers. For privacy reasons, the PINs are anonymised when used in research.

### Study population

The study cohort includes all men and women born in Sweden between 1973 and 1982 who were alive and registered as residents of Sweden at age 18 years (n = 872 912). Due to limited register data on migrants’ circumstances in childhood, residents not born in Sweden were excluded from the cohort. The individuals in the study population were linked to their parents via a Multi-Generation Register.

### Childhood household dysfunction

Indicators of CHDs were selected based upon prior research demonstrating them to have significant adverse health or social implications [10–13, 21–24]. The CHD indicators were all collected from the Swedish population registers listed in Table 1 and they mark events occurring between birth and age 18. All CHD indicators were analysed for both mothers and fathers, and were treated as binary variables (yes/no). Parental alcohol and/or drug misuse was indicated by any entry of alcohol and/or a drug related death or hospital care. Parental mental health problems were indicated by hospital care or cause of death records with a diagnosis related to psychiatric illness and/or self-inflicted injuries. Parental criminality was defined as any criminal conviction leading to a sentence to prison, probation or forensic psychiatric care. These three CHD indicators will also be collectively referred to as ‘parental psychosocial problems’. Having been placed in out-of-home care or in respite care before age 18 was used as a robust indication of child welfare intervention. We also included a variable on household public assistance / social assistance payments as a poverty indicator. Single parent household by age 17 of the child was used as a proxy for parental separation or divorce, which is one of the original variables in the ACE studies. Finally, we included a variable indicating parental death before the child turned 18. Parental death was not included in the original ACE studies but has been shown to be connected to the other indicators and is arguably one of the most stressful events that could happen in a family household [25]. In order to assess cumulative CHD experience, the total number of CHDs was summed and grouped into four categories: zero, one, two and three or more CHD indicator(s).

**Table 1:** Population registers.

Variables	National Register	Years
Date of birth and sex	The Medical Birth Register	1973–1982
Parental PIN	Multi-generation register	1973–1982
Residency in Sweden	Register of the Total Population	1990–1999^1^
Parental socioeconomic position	National Housing and Population Censuses	1985/1990^2^
Parental alcohol/drug misuse	National Patient Register and Cause of Death Register	1973–1999^3^
Parental mental health problems	National Patient Register and Cause of Death Register	1973–1999^3^
Parental criminality	National Register of Criminal Convictions	1973–1999^3^
Child welfare intervention	Swedish Register of Children and Young Persons Subjected to Child Welfare Measures	1973–1999^3^
Single parent household / divorce	Longitudinal Integration Database for Health Insurance and Labour Market Studies (LISA)	1990–1999^1^
Parental death	Cause of Death Register	1973–1999^3^
Household receiving social assistance	Longitudinal Integration Database for Health Insurance and Labour Market Studies (LISA)	1990–1999^1^
Alcohol related illness	National Patient Register and Cause of Death Register	1991–2013^4^ / 2002–2013^5^

^1^ At age 17 of child.

^2^ Census of 1985 was used for the population born 1973–1977, census of 1990 was used for population born 1978–198.

^3^ Age 0–17 of child.

^4^ Alcohol related hospitalisation from 18 years of age.

^5^ Alcohol related outpatient care.

### Alcohol related illness or death

Alcohol related inpatient care was indicated by at least one entry of alcohol related inpatient hospital care (yes/no) in the national patient register. Alcohol related outpatient care was indicated by at least one entry of alcohol related specialised outpatient care (yes/no). Alcohol related death was collected from the cause of death register. The following diagnoses with corresponding ICD-10 codes were used to define alcohol related illness: 'mental and behavioural disorders due to use of alcohol (F10)', 'alcohol fatty liver (K70)', 'alcoholic polyneuropathy (G621)', 'alcoholic cardiomyopathy (I426)', ‘alcoholic gastritis (K292)’. For cases occurring before 1997, the equivalent diagnoses in ICD-9 were used. Alcohol related medical diagnoses that not necessarily imply long-term alcohol misuse, such as alcohol intoxication leading to medical care, were excluded from the measure.

### Socioeconomic position

We categorised the population into six different socioeconomic groups: three levels of non-manual professionals, two levels of manual workers and one unclassified category (‘other’). Childhood SEP was defined by the highest SEP of any adult in the household. This occupation-based SEP measure, similar to the classification discussed by Eriksson [26], has been developed by Statistics Sweden and reflects both level of education and position at the work place [27]. It was measured in the national censuses of 1985 and 1990. The early census was used to measure childhood SEP for the older half of the population and the 1990 census for the other half. Thus the population was between 8 and 12 years old at the time of measurement.

### Statistical analysis

Multivariate analyses were performed using Cox proportional hazard models of time to first alcohol-related inpatient care, outpatient care or death. Entry date was defined as the date of the 18th birthday, and exit date as the date of first hospitalization, outpatient care, date of death, or the end of follow-up (i.e. December 31st 2013). To indicate the strength of the interaction between CHD and socioeconomic background, we calculated a Synergy Index with 95% CI for each outcome [28]. The total number of CHD indicators was reduced to two groups, 0 and 1+. These groups were compared to lowest vs. highest SEP groups. For this analysis, we estimated the interaction between low socioeconomic background (unskilled manual) and any experience of CHD. All analyses were conducted in Stata v11.

### Attrition

Attrition is usually not a major problem in register-based studies. We had no attrition regarding outcome measures or CHD indicators. For 5.1 percent of the population, we had insufficient information regarding parental occupation for categorisation of parental SEP. In the analyses, these cohort members were included in the ‘other’ group.

## Results

Table 2 shows the population stratified into the four CHD categories: experience of zero, one, two and three or more CHD indicators. Experience of CHD was equally distributed in men and women but more common for cohort members with low socioeconomic position. About 50% of those growing up with parents in unskilled manual professions had experience of at least one CHD indicator, the equivalent proportion in the highest socioeconomic group was 25%. The table also lists the individual CHD indicators. Whereas a majority of the population that went through a parental divorce or death had no other experience of CHD, only a small proportion of the population experiencing parental alcohol/drug misuse or child welfare intervention had no other CHD experience. Finally, the table shows the proportion with at least one indication of alcohol related illness or death. Compared to the average of 1.6 percent, the outcome was more common in males, in groups with low socioeconomic background and in all population groups with at least one CHD indicator.

**Table 2:** Childhood household dysfunction, socioeconomic position and alcohol related illness or death.

		Experience of Childhood Household Dysfunction				
	n	0 CHD	1 CHD	2 CHD	3+ CHD	Alcohol related illness or death
**All**	872 912	62.2%	24.7%	8.0%	5.0%	1.6%
Female	420 169	61.7%	25.1%	8.1%	5.2%	1.0%
Male	452 743	62.8%	24.4%	8.0%	4.9%	2.1%
**Childhood socioeconomic position**						
High non-manual	146 356	75.5%	19.8%	3.4%	1.3%	1.1%
Mid non-manual	201 247	71.1%	21.9%	4.8%	2.2%	1.1%
Low non-manual	132 155	62.1%	26.9%	7.3%	3.7%	1.4%
Skilled manual	140 555	63.4%	23.9%	8.3%	4.5%	1.6%
Unskilled manual	154 823	50.1%	29.2%	12.6%	8.2%	2.1%
Other	97 776	42.1%	29.0%	15.0%	13.9%	2.6%
**Indications of childhood household dysfunction**						
Parental alcohol/drug misuse	27 391	-	8.0%	21.5%	70.5%	5.5%
Parental mental health problems	44 004	-	27.2%	31.0%	41.8%	3.4%
Parental criminality	31 519	-	13.1%	28.4%	58.4%	4.9%
Child welfare intervention	36 530	-	7.7%	29.7%	62.6%	7.7%
Divorce/single parent household	254 877	-	62.2%	23.1%	14.7%	2.7%
Parental death	32 024	-	62.0%	21.1%	16.9%	3.0%
Household receiving social assistance	83 249	-	19.5%	42.3%	38.2%	4.2%

Table 3 shows the time-data and the incidence of the alcohol outcomes in the four CHD categories. Incidence rate ratios (IRR) showed that inpatient care was more than nine times more common in the group with cumulative CHD experience (three indicators or more). Although the average follow-up time was about six years shorter for alcohol related outpatient care, this outcome was more common than inpatient care. The gradients of IRRs connected to CHD experience were consistent but tended to be stronger for inpatient care. A total of 604 persons had died due to alcohol related illness. The IRRs for deaths were comparable to those related to alcohol outpatient care. About 0.4 percent of the population had received both inpatient and outpatient care (not shown in tables).

**Table 3:** Incidence of alcohol related illness or death by childhood household dysfunction.

	Follow up period	Person-years	Total number of cases	Incidence rate (95% CI)	IRR
**Alcohol related inpatient care**					
0 CHD	1991–2013 ^a)^	9 882 743	2 289	23.2 (22.2–24.1)	1
1 CHD		3 889 608	1 920	49.4 (47.2–51.6)	2.1 (2.0–2.3)
2 CHD		1 253 987	1 331	106.1 (100.6–112.0)	4.6 (4.3–4.9)
3+ CHD		780 085	1 690	216.6 (206.6–227.2)	9.4 (8.8–10.0)
**Alcohol related outpatient care**					
0 CHD	2002–2013 ^b)^	6 500 835	3 359	51.7 (50.0–53.4)	1
1 CHD		2 573 325	2 688	104.5 (100.6–108.5)	2.0 (1.9–2.1)
2 CHD		831 703	1 639	197.1 (187.8–206.9)	3.8 (3.6–4.0)
3+ CHD		515 025	1 804	350.3 (334.5–366.8)	6.8 (6.4–7.2)
**Alcohol related death**					
0 CHD	1991–2013 ^c)^	9 689 645	213	2.2 (1.9–2.5)	1
1 CHD		3 816 692	178	4.7 (4.0–5.4)	2.1 (1.7–2.6)
2 CHD		1 229 277	99	8.1 (6.6–9.8)	3.7 (2.9–4.7)
3+ CHD		765 474	114	14.8 (12.3–17.8)	6.7 (5.3–8.5)

a) from age 18 to 31 December 2013, average follow-up time is 18.1 years.

b) from 1 January 2002 to 31 December 2013, average follow-up time is 11.9 years.

c) from age 18 to 31 December 2013, average follow-up time is 17.8 years.

The results of the Cox regression analyses are shown in Table 4. The group from the highest socioeconomic background with no experience of CHD is used as the reference group. The table shows that all outcomes were connected to experience of CHD and parental SEP. The results were particularly clear with regard to alcohol related inpatient care. The results for alcohol related outpatient care were similar, although not as pronounced. In the highest socioeconomic group, those experiencing one indicator of CHD had a moderately higher risk for developing alcohol related illness later in life, which is contrasted by the risks in the population experiencing two CHD indicators or three or more. Looking at the population with no experience of CHD, the socioeconomic gradient was quite modest in the analyses. However, there was a substantially higher risk in the groups with low socioeconomic background and experience of CHD. The test for interaction indicated that the combined effect of CHD and socioeconomic disadvantage exceeded the additive effect both with regard to inpatient and outpatient care. The results for alcohol related death were more ambiguous, possibly an artefact of low statistical power (few cases of death). Both socioeconomic disadvantage and experience of CHD implied a risk increase, but there was no interaction effect between the two variables with regard to the outcome.

**Table 4:** Hazard ratios (95% CI) for alcohol related illness and death.

**Alcohol related inpatient care**							
	**Parental socioeconomic position**						
**Numbers of CHD indicators**	**High non-manual**	**Mid non-manual**	**Low non-manual**	**Skilled manual**	**Unskilled manual**	**Other**	Synergy index*
0	1 (ref)	1.1 (1.0–1.3)	1.2 (1.0–1.4)	1.6 (1.4–1.8)	1.8 (1.6–2.1)	1.5 (1.2–1.7)	1.6 (1.4–1.9)
1	2.1 (1.7–2.5)	2.5 (2.1–2.8)	2.5 (2.2–2.9)	2.9 (2.5–3.4)	3.4 (3.0–3.9)	3.2 (2.7–3.7)	
2	5.6 (4.4–7.1)	4.8 (4.0–5.9)	5.8 (4.8–6.9)	5.7 (4.8–6.8)	6.1 (5.3–7.1)	7.0 (6.0–8.2)	
3+	9.4 (7.1–12.4)	9.9 (8.1–12.1)	10.7 (8.9–12.8)	11.7 (9.9–13.8)	12.5 (10.9–14.3)	14.2 (12.4–16.1)	
**Alcohol related outpatient care**							
	**Parental socioeconomic position**						
**Numbers of CHD indicators**	**High non-manual**	**Mid non-manual**	**Low non-manual**	**Skilled manual**	**Unskilled manual**	**Other**	Synergy index*
0	1 (ref)	0.9 (0.8–1.0)	1.0 (0.9–1.1)	1.1 (1.0–1.2)	1.2 (1.1–1.4)	1.1 (0.9–1.3)	1.7 (1.4–2.0)
1	1.7 (1.5–2.0)	1.9 (1.7–2.1)	1.9 (1.7–2.2)	2.2 (1.9–2.4)	2.3 (2.0–2.5)	2.4 (2.1–2.7)	
2	3.3 (2.7–4.1)	3.1 (2.7–3.7)	4.2 (3.6–4.9)	3.5 (3.0–4.1)	4.0 (3.5–4.5)	4.7 (4.1–5.3)	
3+	6.7 (5.3–8.5)	5.8 (4.9–6.9)	6.2 (5.2–7.3)	6.3 (5.4–7.3)	6.8 (6.0–7.6)	8.1 (7.3–9.1)	
**Alcohol related death**							
** **	**Parental socioeconomic position**						
**Numbers of CHD indicators**	**High non-manual**	**Mid non-manual**	**Low non-manual**	**Skilled manual**	**Unskilled manual**	**Other**	Synergy index*
0	1 (ref)	0.8 (0.5–1.2)	0.9 (1.6–1.5)	1.6 (1.0–2.4)	1.3 (0.8–2.0)	1.2 (0.7–2.1)	1.4 (0.8–2.6)
1	2.2 (1.3–3.8)	2.3 (1.4–3.7)	2.3 (1.4–3.7)	2.6 (1.6–4.3)	2.5 (1.6–3.9)	2.2 (1.3–3.7)	
2	2.4 (0.9–6.7)	2.8 (1.4–5.8)	2.7 (1.3–5.6)	3.8 (2.0–6.9)	5.6 (3.6–8.8)	5.3 (3.2–8.7)	
3+	10.7 (4.8-24-1)	3.9 (1.7–9.3)	7.9 (4.3–14.6)	11.1 (6.6–18.8)	6.5 (4.0–10.5)	7.7 (4.8–12.1)	

* Comparing the highest and the lowest SEP groups and a dichotomous measure of CHD (no experience/any experience).

A sensitivity analysis was conducted to make sure that the results were not entirely explained by biological heredity. Table 5 shows this sensitivity analysis using the same study population, but excluding individuals with indications of parental alcohol/drug misuse (n = 27 391) and parental mental health problems (n = 44 004), with the hazard ratios connected to the indicators of CHD. The exclusion of these individuals did not lead to any great alteration of the association between CHD and alcohol related illness and death in young adulthood, but the HRs remained more or less similar to the results presented in Table 4.

**Table 5:** Hazard ratios (95% CI) for alcohol related illness and death excluding cases of parental alcohol misuse and parental mental health problems.

**Alcohol related inpatient care**							
	**Parental socioeconomic position**						
**Numbers of CHD indicators**	**High non-manual**	**Mid non-manual**	**Low non-manual**	**Skilled manual**	**Unskilled manual**	**Other**	**Synergy index**
0	1 (ref)	1.1 (1.0–1.3)	1.2 (1.0–1.4)	1.6 (1.4–1.8)	1.8 (1.6–2.1)	1.5 (1.2–1.7)	1.6 (1.4–1.9)
1	2.1 (1.8–2.5)	2.5 (2.2–2.9)	2.5 (2.2–2.9)	3.0 (2.6–3.5)	3.4 (3.0–3.9)	3.2 (2.7–3.8)	
2	5.7 (4.3–7.6)	5.3 (4.3–6.6)	6.2 (5.0–7.5)	6.1 (5.1–7.3)	6.6 (5.6–7.6)	7.1 (6.1–8.4)	
3+	8.9 (5.1–15.4)	12.4 (9.1–16.7)	14.5 (11.1–18.9)	15.7 (12.6–19.6)	13.4 (11.2–15.9)	13.8 (11.7–16.4)	
**Alcohol related outpatient care**							
	**Parental socioeconomic position**						** **
**Numbers of CHD indicators**	**High non-manual**	**Mid non-manual**	**Low non-manual**	**Skilled manual**	**Unskilled manual**	**Other**	Synergy index*
0	1 (ref)	0.9 (0.8–1.0)	1.0 (0.9–1.1)	1.1 (1.0–1.2)	1.2 (1.1–1.4)	1.1 (0.9–1.3)	1.7 (1.3–1.9)
1	1.7 (1.5–2.0)	2.0 (1.7–2.2)	2.0 (1.7–2.2)	2.2 (2.0–2.5)	2.3 (2.0–2.5)	2.4 (2.1–2.7)	
2	3.5 (2.7–4.5)	3.6 (3.0–4.4)	4.3 (3.7–5.1)	3.5 (2.9–4.1)	4.1 (3.6–4.7)	4.9 (4.3–5.6)	
3+	8.0 (5.2–12.2)	6.5 (4.9–8.9)	6.9 (5.3–9.0)	7.5 (6.0–9.4)	7.1 (6.1–8.4)	7.9 (6.8–9.1)	
**Alcohol related death**							
	**Parental socioeconomic position**						
**Numbers of CHD indicators**	**High non-manual**	**Mid non-manual**	**Low non-manual**	**Skilled manual**	**Unskilled manual**	**Other**	**Synergy index**
0	1 (ref)	0.8 (0.5–1.2)	0.9 (0.6–1.5)	1.6 (1.0–2.4)	1.3 (0.8–2.0)	1.2 (0.7–2.1)	1.5 (0.7–2.9)
1	2.3 (1.4–4.0)	2.4 (1.5–3.8)	2.2 (1.3–3.6)	2.8 (1.7–4.5)	2.6 (1.6–4.0)	2.1 (1.2–3.6)	
2	2.1 (0.5–8.8)	4.0 (1.9–8.6)	3.7 (1.7–7.8)	5.2 (2.8–9.6)	6.8 (4.2–10.8)	5.7 (3.4–9.7)	
3+	20.9 (6.4–67.6)	5.3 (1.3–22.0)	13.0 (5.5–30.6)	10.2 (4.3–24.1)	6.1 (2.9–12.6)	10.7 (6.1–18.8)	

* Comparing the highest and the lowest SEP groups and a dichotomous measure of CHD (no experience/any experience).

## Discussion

In line with previous research, this national cohort study of over 870,000 Swedish men and women confirms our first hypothesis by showing a clear and cumulative connection between CHD and alcohol related illness and death in young adulthood. CHD also interacted with parental SEP in the way that the risks connected to cumulative CHD experience and low socioeconomic background added up to a high risk increase for this group compared to the population with high socioeconomic background and no experience of CHD.

Explanations from various disciplines may help us understand our results. *Biological approaches* need to be taken into account when considering the hereditary components linking parental and offspring alcohol misuse as well as parental and offspring mental health problems. Furthermore, as these problems are interrelated, parental mental health problems may be transferred to the child, which in turn increases the risk for unhealthy alcohol consumption in the offspring later in life [11, 29]. In order to ensure that the association was not entirely explained by the intergenerational transfer of alcohol misuse or mental health problems, we conducted a sensitivity analysis where we excluded individuals with indications of parental alcohol/drug misuse and parental mental health problems. In other words, the sensitivity analysis included only the population with no CHD or any CHD unrelated to parental alcohol/drug misuse or parental mental health problems. The association with offspring alcohol related illness in young adulthood remained strong in this analysis. *Psychological explanations* often focus on the adverse effect that a childhood trauma, such as parental death, parental divorce or parental psychosocial problems, will have on long-term health [15, 23]. Alcohol may be used as a way to self-medicate against the mental health problems that may follow these kinds of experiences [24]. The fact all socioeconomic groups are adversely affected by CHD speaks to the universally harmful potential of these experiences. However, our results also suggest that *sociological approaches* are important to take into account in studies on CHD and health later in life. The socioeconomic gradient found in this study was weaker than what has been found in previous studies, which suggests that parts of the socioeconomic differences are explained by a higher prevalence of CHD in low socioeconomic groups [3]. The fact that records of CHD were more common in this population is in itself not surprising. Firstly, some of the indicators, such as child welfare interventions or social assistance, are directly linked to the relatively disadvantaged situation in lower socioeconomic groups [30, 31]. Also, a consequence of using the highest SEP of any parent as a measure of household SEP is that the only possible socioeconomic mobility after a divorce is downwards, which often reflects the reality, especially for many newly separated women [32]. Secondly, it might be that the possibility to hide psychosocial problems from public intervention (and thus from the registers) is greater among parents with high SEP as they may have alternative resources to help them cope with their problems. In other words, parental psychosocial problems such as alcohol misuse may have different consequences depending on the socioeconomic context [33]. Thirdly, a social selection mechanism is possible, with parents having psychosocial problems being more likely to be unemployed or in occupations requiring less formal qualification [34].

A life course perspective is helpful in order to understand the combined effect that CHD and a socioeconomically disadvantaged background may have on alcohol related illness and death later in life. Economic strains and parental psychosocial problems during pregnancy are associated with maternal stress, nutritional deficiencies and insufficient prenatal care, which may lead to a less than optimal foetal development setting the conditions for the child at time of birth [35, 36]. During childhood and adolescence, household poverty, parental psychosocial problems, and traumatic events may affect the child’s long-term health directly and indirectly. Poor parental care and psychosocial pressures may put chronic burdens on the child during the formative years. The stressful situation related to poverty in combination with psychosocial problems (e.g. substance misuse) may make the parents less able to provide the children with a cognitively stimulating environment. This may impact the school performance and the education of the child, which have been shown to be strongly associated to alcohol related illness and death later in life [37]. Traumatic events like parental deaths, or in some cases divorces, are immediate stressors that together with the aforementioned factors add up to a cumulative exposure with the potential to make the child more vulnerable to the adverse effects of alcohol later in life.

## Strengths and Limitations

The large population size in combination with the detailed register data is a major strength of this study. We were able to identify a number of CHD indicators in the registers and link them to all alcohol related diagnoses found in the National Patient Register and to socioeconomic variables. The significant population size allowed for detailed analyses demonstrating the cumulative effect of CHD and how it interacts with socioeconomic background. Other quantitative studies with a similar design and research questions, but with other kinds of data sources often struggle with insufficient power and missing data that due to the sensitive nature of the topic might be substantial. In our case, attrition was negligible.

The use of population registers in studies of alcohol related illness and death also come with some limitations. A major concern is the proportion of hidden alcohol misuse and hidden indicators of household dysfunction that are both likely to be high, potentially more so in some population groups. For example, a high socioeconomic position might provide the families and the individuals with resources enabling them to cope with household problems and alcohol misuse without assistance from society (e.g. hospital care or child welfare interventions), with the consequence that they do not end up in registers. Also, although the national registers provide valuable information, they do not include any data on exposure to abuse or neglect, which made it difficult to replicate any of the original ACE studies. There is also some potential risk for referral bias by the medical doctors diagnosing the patients. It could be more likely that alcohol related diagnoses are given to individuals reflecting the stereotype of an alcohol addict. Conditions that are not immediately associated with alcohol misuse, such as gastritis, cardiomyopathy or polyneuropathy may be misclassified due to the patient’s social background. The fact that we limited the study to native-born Swedes makes the study unrepresentative of all Swedish residents, given the large migrant population. Also the specifics of the Swedish welfare system should be considered before applying these results to other contexts. These limitations highlight the importance to use a spectrum of data materials and research methodologies when studying this subject. Survey-based studies and qualitative research have better opportunities to account for important information that remain hidden in the population registers.

## Implications for Research, Policy and Practice

Our study gives further support to the research underlining the importance of childhood environment for health later in life. In addition to Sweden’s general public health target of good and equal health in the entire population [38], the Swedish government now aims to close the health gap within one generation [39]. This ambitious goal calls for radical action. Giving support to children growing up in dysfunctional households should be part of any strategy aiming for population health equity. Future studies should look more closely at which kind of support would be particularly beneficial.

## Conclusion

Childhood household dysfunction had a strong and cumulative connection to alcohol related illness in young adulthood. Experiences of CHD had adverse effects in all socioeconomic groups, but the combination of low socioeconomic background and CHD had a particularly strong relationship to alcohol related illness in young adulthood. Not only is CHD a strong risk indicator for adult ill health, but as this study has shown, it is also connected to socioeconomic disadvantage. This may call for a dual strategy to target both CHD and childhood socioeconomic inequalities as both have a great potential to decrease the intergenerational transfer of social inequalities in health.
